# A Systematic Review and Comparative Analysis of Reconstructive Rhytidectomy: Advantages, Disadvantages, and Patient Outcomes

**DOI:** 10.7759/cureus.71006

**Published:** 2024-10-07

**Authors:** Christopher R Meretsky, Paulette Hausner, Brian P Flynn, Anthony T Schiuma

**Affiliations:** 1 Surgery, St. George's University School of Medicine, Great River, USA; 2 Obstetrics and Gynecology, St. George's University School of Medicine, Great River, USA; 3 Medicine, St. George's University School of Medicine, Great River, USA; 4 Orthopedic Surgery, Holy Cross Hospital, Fort Lauderdale, USA

**Keywords:** aesthetic surgery, complications, facelift, minimally invasive, patient outcomes, patient satisfaction, reconstructive rhytidectomy, surgical techniques

## Abstract

Reconstructive rhytidectomy, commonly known as facelift surgery, is a prominent cosmetic procedure aimed at rejuvenating facial appearance by addressing signs of aging. This paper critically evaluates the advantages and disadvantages of various surgical techniques involved in rhytidectomy, including the superficial musculoaponeurotic system (SMAS), deep plane facelift, and subperiosteal approaches. This systematic review of recent literature highlights key outcomes such as scar quality, postoperative pain management, and patient satisfaction. While the techniques demonstrate significant improvements in aesthetic results and patient quality of life, they also present risks including complications, dissatisfaction with outcomes, and the financial burden of surgery. Future directions indicate a trend toward minimally invasive approaches, integration of regenerative medicine, and personalized surgical planning, aiming to optimize results and minimize risks.

## Introduction and background

Reconstructive rhytidectomy, also known as facelift, is one of the most sought-after cosmetic procedures worldwide, offering individuals the opportunity to rejuvenate their appearance by addressing the visible signs of aging [1]. The procedure typically involves the repositioning and tightening of facial tissues, the removal of excess skin, and, in some cases, the incorporation of fat grafting or other volumetric enhancements to restore a youthful contour [2]. Although rhytidectomy has gained significant popularity and has seen remarkable advancements in surgical techniques, it still carries inherent risks and challenges that cannot be overlooked. Patients considering this procedure should be aware of potential complications and the importance of selecting a qualified surgeon to ensure the best possible outcomes [3].

Reconstructive rhytidectomy, or facelift surgery, involves a range of surgical techniques tailored to restore facial contours and address the effects of aging or trauma [4]. Key methods include the superficial musculoaponeurotic system (SMAS) technique, deep plane facelift, and subperiosteal approaches [5]. The SMAS technique focuses on lifting and repositioning the underlying facial musculature and tissues, while the deep plane facelift addresses deeper tissue layers for more dramatic results with longer-lasting effects [6]. The subperiosteal approach targets the facial skeleton and soft tissue attachments for comprehensive rejuvenation. Each technique is selected based on individual patient anatomy, desired outcomes, and the surgeon's expertise, aiming to enhance aesthetic appearance and function while minimizing scarring and recovery time.

The complexity of the procedure demands a high level of surgical expertise, as well as a thorough understanding of facial anatomy and the aging process [7]. Factors such as skin type, age, and individual patient goals all play a critical role in determining the most appropriate surgical approach. Additionally, the risk of complications, including scarring, hematoma, nerve injury, and dissatisfaction with aesthetic outcomes, underscores the importance of meticulous preoperative planning and postoperative care [8].

This paper aims to critically evaluate the advantages and disadvantages of reconstructive rhytidectomy by reviewing the latest surgical techniques and methodologies that have been developed to enhance patient outcomes. Through a comprehensive analysis of recent data on scar quality, postoperative pain management, and patient satisfaction, this study seeks to provide valuable insights into the current state of reconstructive rhytidectomy. Furthermore, it explores future directions for the procedure, with a focus on optimizing results and minimizing risks, ultimately contributing to the ongoing evolution of facial rejuvenation surgery.

## Review

Methods

Study Selection

**Figure 1:**
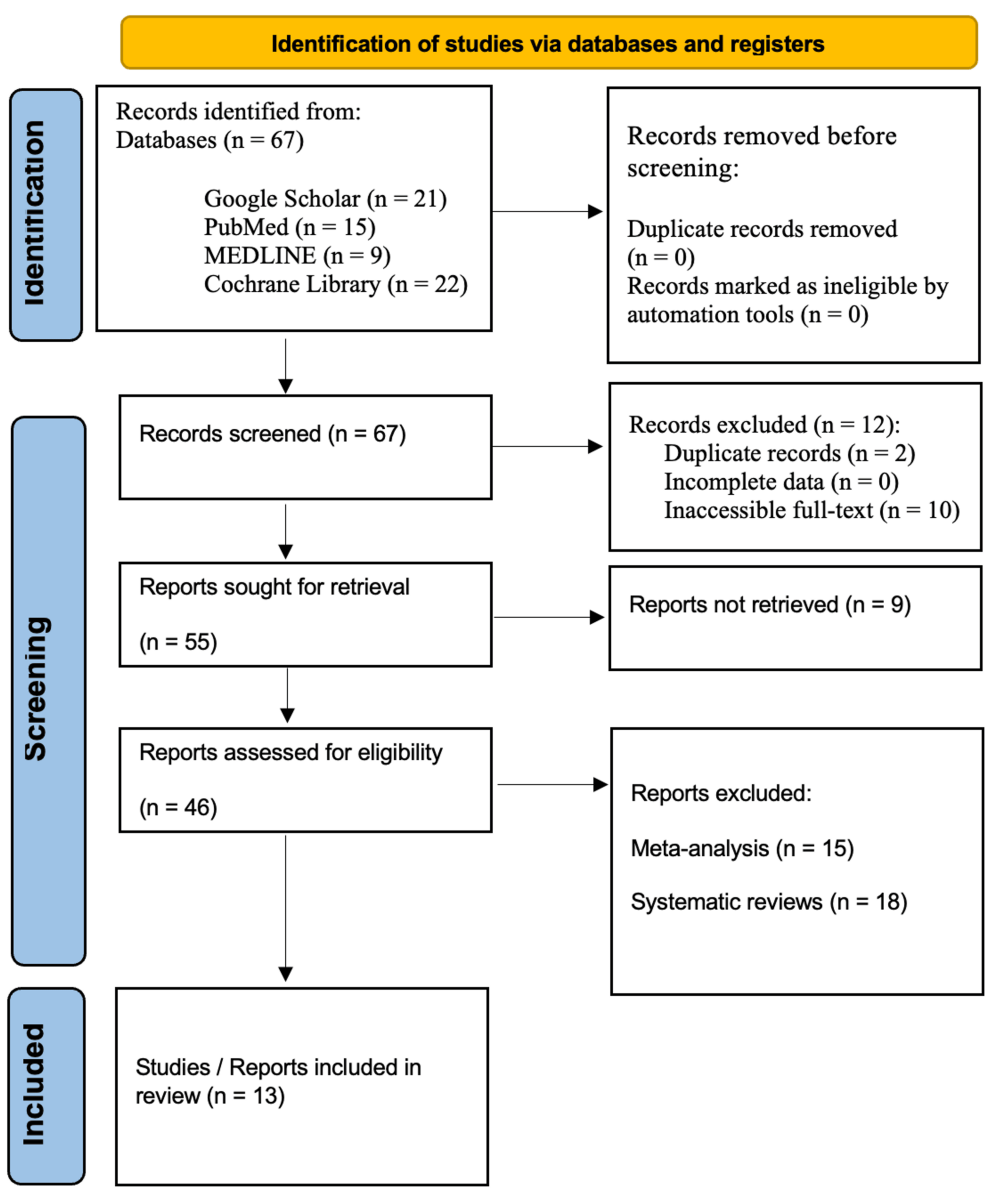
PRISMA flowchart: literature search and study selection n, number; PRISMA, Preferred Reporting Items for Systematic Reviews and Meta-Analyses Source: [9]

In line with the Preferred Reporting Items for Systematic Reviews and Meta-Analyses (PRISMA) guidelines, a systematic review was conducted. Comprehensive searches were performed in the databases of Google Scholar, PubMed, MEDLINE, and the Cochrane Library for studies published over the last two decades, specifically from 2004 to 2024. The search utilized targeted keywords including "Reconstructive OR facelift Rhytidectomy," "Surgical techniques in Reconstructive Rhytidectomy OR facelift," "Advantages of Reconstructive Rhytidectomy OR facelift," "Disadvantages of Reconstructive Rhytidectomy OR facelift," and "Reconstructive Rhytidectomy OR facelift and future directions." The review adhered to the PRISMA guidelines to ensure transparency and reproducibility throughout the research process (Figure 1).

Inclusion Criteria

The studies eligible for inclusion in this review had to satisfy specific criteria. Firstly, they needed to involve human participants undergoing reconstructive rhytidectomy. Secondly, the studies had to report on scar quality. Additionally, they were required to present outcomes related to various factors, including the level of postoperative pain experienced by patients and their overall satisfaction. Lastly, the studies must be published in English.

Exclusion Criteria

We excluded specific studies from our selection criteria. In particular, studies that did not offer adequate data on reconstructive rhytidectomy cases were left out. We also omitted meta-analyses, reviews, or editorials that did not present original findings. Furthermore, research based solely on animal models was excluded. This thorough selection process was designed to improve the relevance and reliability of our review by focusing exclusively on primary studies that directly relate to the human population of interest.

Patient Selection and Classification

The review included patients who underwent rhytidectomy for both cosmetic and reconstructive purposes, emphasizing the diverse motivations behind these surgeries. To facilitate a more granular analysis, we classified patients based on the specific surgical techniques employed. This classification included various approaches, such as the SMAS facelift, deep plane facelift, and mini facelift, allowing for a focused examination of outcomes associated with different methodologies.

Outcome Measures

Our review concentrated on several essential outcome measures. We evaluated the quality of scars using validated assessment tools, such as the Patient and Observer Scar Assessment Scale (POSAS), to provide objective insights into scarring outcomes. Additionally, we assessed the quality of postoperative pain through standardized pain measurement scales, including the Visual Analog Scale (VAS), to gauge patient discomfort accurately. Lastly, we examined postoperative satisfaction rates by utilizing patient surveys and validated satisfaction scales, ensuring a comprehensive understanding of patients' experiences following their surgeries. This framework of outcome measures facilitated a thorough evaluation of the effectiveness and patient perception of reconstructive rhytidectomy procedures.

Results

Table 1 summarizes the last 20 years of clinical studies targeting patients who underwent rhytidectomy (facelift) for reconstructive purposes. A total of 13 studies were reported, which included nine observational studies, three case reports, and one randomized clinical trial. These studies have highlighted several innovative and advanced facelift techniques that have emerged in recent years. One of the key advancements was the use of tissue sealants, such as Artiss (Baxter) and fibrin, which help enhance tissue adhesion and promote wound healing [10-23]. Power-assisted dissection, a technique that utilizes specialized surgical tools to facilitate tissue separation, was also reported. The deep plane facelift, a surgical approach that targets the deeper layers of the face, was another innovative technique described in the literature. Furthermore, the paragraph mentions the use of composite face allotransplantation, a highly complex procedure where a portion of the face is transplanted from a donor to the recipient. The ponytail lift, a less invasive facelift technique that utilizes the natural contours of the hairline, and the mini-invasive facelift, which aims to minimize scarring, were also reported. Other advancements included the use of autologous fibrin glue or platelet-poor plasma, suction drainage to prevent fluid accumulation, the stepped lift of the superficial musculoaponeurotic system (SMAS), and the extended deep plane rhytidectomy, which targets the deeper facial structures [24]. These studies highlight the rapid evolution of facelift techniques, driven by the ongoing pursuit of improved surgical outcomes, reduced complications, and enhanced patient satisfaction. As the field of facial rejuvenation continues to advance, these innovative approaches may hold the potential to deliver more effective and personalized treatment options for patients undergoing reconstructive facelift procedures.

**Table 1:** Summary of clinical studies on reconstructive rhytidectomy techniques, outcomes, and considerations over the past 20 years (2004-2024) DM, diabetes mellitus; SHT, systemic hypertension; SMAS, superficial musculoaponeurotic system Source: [10-22]

References	Study design	Patient age and gender	Pathological anomaly presented	Surgery technique(s)	Patient's medical history	Postoperative results	Advantages	Disadvantages
Wirth et al. [10]	Case report	A female in her mid-30s	Oculopharyngeal muscular dystrophy	-	Alopecia totalis of unknown origin, low blood pressure, and Graves' disease believed to be secondary to thyroiditis. She was prescribed Synthroid, Prozac, Estrace, and Provera. For pain management, she frequently took Percocet and used Atarax at night to aid sleep. She took Prilosec, urecholine, and Propulsid to address gastrointestinal symptoms.	The patient made a remarkable recovery, with no complications arising from the surgery or the recovery period. Just days after the procedure, she was able to discontinue some pain medications that she had been taking chronically for several years.	There were no complications related to the surgery or the recovery period. Within days after the procedure, the patient was able to discontinue some pain medications that she had been taking chronically for several years.	No disadvantages reported
García-Díez et al. [11]	Observational study	3 males and 4 females aged 28-50 years of age	Lateral mandibular defects due to the resection of benign mandibular tumors	A combined rhytidectomy technique utilizing an intraoral incision was employed for mandibular resection and the reconstruction of defects with vascularized free osseous flaps.	-	The iliac crest was utilized for reconstruction in six cases, while one patient received a fibula graft. Two patients experienced transient paresis of the marginal nerve, and one patient suffered flap loss, necessitating repeat microvascular surgery. Dental rehabilitation with osseointegrated implants was conducted for four patients. All patients achieved successful mandibular reconstruction, with functional outcomes rated as excellent for everyone.	Provides excellent access to the posterior mandible and facilitates predictable identification of neural and vascular structures; associated with minimal morbidity compared to traditional neck incisions; achieves a natural facial contour, perfect symmetry, and an inconspicuous scar; allows for microvascular mandibular reconstruction in a single stage, enabling quicker recovery for patients. The technique preserves the original attached gingiva, which is beneficial for dental rehabilitation.	The surgical field can be restricted, especially when recipient vessels are deeper and located in a lower cervical area; is not indicated for cases requiring extensive extramandibular margins during tumor resection. There is a risk of complications such as transient facial paralysis and flap loss, although these are generally manageable. It may demand a high level of skill and experience from the surgical team to achieve optimal outcomes.
Yamamoto et al. [12]	Observational study	120 patients, with a female-to-male ratio of 54:6 in the Tisseel group and 55:5 in the Artiss group. The average ages for the Tisseel and Artiss groups were 63 and 60 years, respectively.	Hematoma, fluid accumulation necessitating needle aspiration, seroma, flap necrosis, infection, and nerve damage	Artiss (Baxter) and fibrin tissue sealants	-	In the study involving tissue sealants, the Tisseel group reported two cases of fluid collection that required needle aspiration, with no other complications noted. In contrast, the Artiss group experienced 10 complications, including nine fluid collections needing aspiration and one hematoma. Among 179 respondents, 34% reported using tissue sealants in rhytidectomies, while 66% did not. Artiss is effective and safe for rhytidectomies, eliminating the need for surgical drains, with a complication rate comparable to Tisseel.	Fibrin: sealant included the elimination of the need for surgical drains (59%), ease of use (47%), a reduction in hematoma, edema, or fluid accumulation (47%), and simplified postoperative care (41%).	Fibrin: sealant included higher costs (90%), the risk of infection or allergic reactions (33%), the need for education of operating room staff (33%), and limited availability (23%).
Jacono et al. [13]	Observational study	300 hemifaces were operated on, consisting of 147 females and 3 males. The average age of the patients was 60 years.	Patients seeking surgery for functional problems, including facial paralysis or other reconstructive reasons, were not included.	Extended deep plane rhytidectomy	The duration between previous aesthetic facial surgeries and the revision rhytidectomy for patients was recorded.	The mean resultant angle for the cohort was measured at 60° from the horizontal, with a range of 46°-77°. An inverse correlation was observed between the angle and patient age (r = –0.3). Younger patients (under 50 years) exhibited a significantly more vertical angle of 64° compared to older patients (70 years and above), who had an angle of 56°. There were no significant differences between the hemifaces of different subjects.	A new technique was introduced for determining the angle of maximal rejuvenation during rhytidectomy. In all cases, this angle was found to be more superior than posterior and is closely associated with the patient's age. Achieving lasting results requires a thorough anatomical understanding and meticulous attention to both the direction and degree of skin laxity.	No disadvantages reported
Zhou et al. [14]	Observational study	54 patients; all were female, aged 44-69 years	Forehead wrinkles, crow's feet, fullness in the upper cheeks, fullness in the lower cheeks, nasolabial folds, and jawline definition	Stepped lift of the superficial musculoaponeurotic system	-	The overall complication rate for the current surgical approach was 7.40%. This technique led to reduced operating time and drainage volume compared to previous methods. All patients were satisfied with their results, feeling they looked 8.3 years younger than their actual age. The highest satisfaction was noted in the midface, temple, and nasolabial folds, with significant improvements evident in pre- and postoperative photographs.	The modified facelift procedure, along with the stepped superficial musculoaponeurotic layer lift technique, proved effective for elderly patients with significant facial wrinkles and ptosis, delivering a long-lasting, natural, and youthful appearance.	No disadvantages reported
Rezende et al. [15]	Randomized controlled trial	72 female patients aged 43-79 years	Signs of facial aging, including flaccidity, ptosis, and wrinkles, indicating a medical need for rhytidoplasty for aesthetic reasons	Autologous fibrin glue/platelet-poor plasma and suction drainage	Smoking, DM, and SHT	The average total volume of exudate was 3.21 mL in the suction drainage group compared to 1.02 mL in the fibrin glue group, yielding an effect size of 68.1% with a confidence interval of 55.3-77.2. The results strongly support the use of fibrin glue, demonstrating that it was 68.1% more effective than suction drainage in preventing hematoma or seroma during rhytidectomy procedures.	-	-
Rohrich et al. [16]	Observational study	83 male and 83 females; the mean age was 59 years	Hematoma, seroma, skin sloughing, alopecia, infection, and nerve injury	Facelift flap	Hypertension	The study emphasizes the differences in facial analysis and rhytidectomy techniques between male and female patients. In the control group, five male patients (6%) experienced postoperative hematoma, while no female patients were affected. There were no other complications reported.	By considering the natural patterns of hair growth in incision design, ensuring that follicles are not harmed during dissection, and adhering to standard care practices to prevent postoperative hematomas, it has achieved safe, consistently reproducible aesthetic results with acceptable rates of complications.	No disadvantages reported
Saha [17]	Observational study	10 patients (8 males and 2 females) aged between 21 and 78 years	Without complications	Facelift flap	Superficial necrosis occurring in one patient (10%) and mild venous stasis in three patients (30%)	The augmented facelift flap effectively reconstructed defects in the medial cheek, lateral orbit, and lower eyelid, yielding aesthetically pleasing outcomes. Of the patients, nine (90%) healed without long-term complications. Short-term complications included superficial necrosis in one patient (10%) and mild venous stasis in three patients (30%), with no cases of flap loss or infection reported. Overall, patient satisfaction scores ranged from 50% to 90%, with an average of 78%.	The augmented facelift flap effectively reconstructed post-traumatic facial defects in the cheek, lateral orbit, and lower eyelids, achieving satisfying aesthetic outcomes.	No disadvantages reported
Campo [18]	Observational study	672 patients, along 19 years	Temporary hypoesthesia may occur in the forehead or cheeks, along with occasional temporary palsy of the frontotemporal branch of the facial nerve.	Mini-invasive facelift	-	The results are more long-lasting because this technique alleviates the weight exerted by the descent of deep tissues against the skin, enabling it to regain some of its lost elasticity. These procedures are highly safe and pose fewer risks compared to other deep plane facelift techniques.	This technique helps restore the facial structure, enhance cheekbone prominence, redistribute lax skin, and indirectly tighten the skin.	No disadvantages reported
Kao et al. [19]	Observational study	600 patients	-	Ponytail lift	-	There were no occurrences of postoperative skin flap necrosis, and no permanent nerve injuries were reported. In 20 cases, an additional surgical touch-up procedure was conducted to address unmet aesthetic needs.	This approach offers a deep plane facelift without the burden of visible scars, as the incisions are concealed within the temple scalp, post-auricular area, and posterior scalp. The techniques outlined are both safe and effective, delivering reliable and satisfying results.	No disadvantages reported
Siemionow et al. [20]	Case report	A 45-year-old female	Her facial deformities comprised the absence of the nose, nasal lining, and underlying bone; contracted remnants of the upper lip; loss of function in the orbicularis oris and orbicularis oculi muscles; distorted and scarred lower eyelids deficit, resulting in impaired midface function.	Composite face allotransplantation	With severe midface trauma, she underwent a near-total face transplantation, during which 80% of her face was replaced with a customized composite tissue allograft.	The rejection was successfully reversed with a single bolus of corticosteroids. In the initial 3 weeks following the transplantation, the patient adapted to her new face. Six months after the surgery, her functional outcome has been outstanding. Compared to her condition prior to the procedure, she can now breathe through her nose, enjoy her sense of smell and taste, speak clearly, consume solid foods, and drink from a cup.	The viability of reconstructing severely disfigured patients through a single surgical procedure utilizing composite face allotransplantation was demonstrated.	No disadvantages reported
Gadallah et al. [21]	Case report	50 female patients between 30 and 56 years of age	-	Deep plane facelift	-	Patient and surgeon satisfaction with postoperative cosmetic outcomes was evaluated using a proforma scale, where scores ranged from 1 for poor results to 5 for excellent results.	A high score of both satisfaction grades was perceived.	During the follow-up, we observed four cases of seroma, four instances of facial mandibular nerve neuropraxia, five cases of scar hypertrophy, two occurrences of wound infection, and one case of pixie ear deformity.
Cohen et al. [22]	Observational study	34 patients (31 females and 3 male), aged 50-77 years	Minor epidermolysis occurred at the junction of the postauricular incision and the hairline; significant laxity and retroauricular hematoma	Facelift with power-assisted dissection	Photodamage, subcutaneous deep and superficial fat compartment loss and bone loss and/or pre-existing skeletal deficiency, and laxity	The dissection of skin flaps and SMAS elevation were completed more quickly than with traditional techniques. There was a notable reduction in bleeding. The skin flaps showed improved perfusion, exhibiting less venous engorgement and ecchymosis compared to those created with sharp scissor dissection. Overall, patients experienced shorter postoperative recovery times and reduced social downtime due to less bruising and edema.	The duration of the procedure has been shortened, and patients have encountered less social downtime compared to our previous experience with scissor dissection.	-

The cosmetic techniques that were reviewed demonstrated several key advantages for patients. Multiple studies found that individuals who underwent the procedures reported very high levels of satisfaction on post-operation surveys, indicating that the treatments helped significantly improve their quality of life. Clinical trials determined that the techniques were both safe and effective when performed by skilled surgeons. The procedures reliably delivered satisfying aesthetic results for patients while also posing very little risk of adverse outcomes. Specifically, the techniques helped restore facial structure and enhance cheekbone prominence by tightening underlying tissues and muscles. They also helped redistribute excess or lax skin on the face, cheeks, and neck areas. The techniques tightened facial skin in a way that did not harm or damage hair follicles, preventing issues such as poor wound healing or hair loss [25]. Surgeons found that a modified facelift procedure, when combined with a stepped superficial musculoaponeurotic layer (SMAS) lift technique, proved highly effective for elderly patients experiencing significant facial wrinkling and sagging of skin and tissues. These composite procedures provided patients with a long-lasting, natural, and youthful appearance without the visible signs of aging that can make people feel self-conscious or older than their actual age. The overall participant satisfaction with the cosmetic results remained very high years after their procedures.

While the techniques demonstrated several clear benefits, the review also reported some potential minor disadvantages and risks that should be carefully considered. Specifically, a small number of cases of seroma formation (fluid buildup in tissues), facial mandibular nerve neuropraxia (temporary nerve injury or weakness), and scar hypertrophy (enlarged or raised scarring) were reported. Infrequent wound infections and occasional pixie ear deformities (abnormally pointed or prominent ears) were also noted, requiring further interventions in rare cases. There remained inherent risks associated with any surgical procedure, such as infection, allergic reactions to medications or dressings, and transient facial paralysis that usually resolved within a few weeks. However, instances of full-thickness flap loss were exceptionally uncommon and generally manageable with revision procedures, if needed [26]. The operative field could sometimes be restricted for surgeons, particularly when recipient blood vessels for tissue transfers were located deeper in the lower cervical area of the neck. This increased the technical challenge of the procedures in some individuals. However, experienced cosmetic surgeons found ways to safely overcome these difficulties in most situations. Overall, the disadvantages proved minor when weighed against the substantial benefits patients gained from the aesthetic and quality of life improvements. With proper patient selection and surgical skills, the risks remained very low.

Upon reviewing the available literature on these techniques, several key conclusions could be drawn regarding their effectiveness and outcomes. Overall, the modified facelift procedures and composite SMAS lifts demonstrated promising results in achieving highly satisfactory aesthetic outcomes for patients undergoing rhytidectomy, or a facelift procedure, specifically for reconstructive purposes rather than solely cosmetic reasons [27]. Clinical studies and case reports found that the techniques reliably corrected issues such as ptosis of facial tissues, redistributed loose or excess skin, tightened underlying muscular structure, and restored youthful contouring when performed by skilled plastic surgeons. Not only did participants report high levels of satisfaction on follow-up surveys regarding their cosmetic results, but independent evaluators also judged photographic documentation as showing natural and long-lasting rejuvenation without obvious signs of procedural manipulation. For those seeking reconstruction after facial trauma, weight loss, aging, or similar conditions causing structural deformities, the reviewed techniques restored lost definition, proportion, and balance to features. This validated their usefulness not just for aesthetic goals but for functional reconstruction as well. Additionally, follow-up appointments found that results generally continued improving even months after surgery as residual swelling subsided. In conclusion, the modified facelift procedures and composite SMAS lifts proved effective in attaining cosmetically pleasing and psychologically gratifying outcomes for patients undergoing facelifting for rejuvenative or reconstructive needs.

Discussion

Over the past few decades, facial aesthetic surgery has experienced remarkable growth, both in terms of quantity and quality. This evolution has been driven by advancements in surgical techniques and treatment strategies, stemming from a deeper understanding of facial anatomy and the aging process. Among the various procedures, facelift has undergone significant innovation over the years. Remarkably, despite these advancements, facelift itself is just over a century old [23].

A recent study that aimed at reconstructing defects resulting from skin tumor resections presented the use of laser surface scanning to evaluate facial symmetry following unilateral facelift procedures. The research involved six patients who underwent defect reconstruction using flaps raised from the subcutaneous layer. Immediate postoperative photographic assessments revealed facial asymmetry due to unilateral skin tension. However, after a minimum follow-up of one year, both photographic and laser surface scanning analyses indicated restoration of facial symmetry. Researchers concluded that laser surface scanning emerges as a promising technology for objectively assessing outcomes and could be utilized to evaluate both immediate and long-term effects of rhytidectomy procedures. Subcutaneous flaps, without the need for duplication or resection of the superficial musculoaponeurotic system, are particularly suitable for unilateral procedures, as they effectively restore facial symmetry within one year [24].

A diverse range of rhytidectomy techniques has been documented, varying from skin-only procedures to those involving manipulation of the superficial musculoaponeurotic system (SMAS). Techniques that enable the SMAS to support the weight of the subcutaneous tissue and serve as the primary element in contouring may lead to longer-lasting results [25]. O'Connell introduces an innovative method for minimal incision rhytidectomy alongside a traditional rhytidectomy, employing bidirectional barbed sutures to create a two-layer SMAS plication. This technique minimizes the risks of bleeding and nerve damage while ensuring that knots are not visible, palpable, or prone to extrusion. In numerous instances, the standard bidirectional lift can be conducted under local anesthesia, with or without slight oral sedation [26]. The contemporary facelift is a blend of multiple techniques, evolving from a one-size-fits-all method to a focus on personalized component analysis. By customizing the procedure for each patient, optimal outcomes can be attained in both primary and secondary rhytidectomy procedures [27].

Advantages

Reconstructive rhytidectomy provides numerous benefits for individuals aiming to enhance their facial appearance and regain a youthful look. One of the main advantages is the significant reduction of sagging skin and wrinkles, which can boost self-esteem and confidence. This procedure not only tightens the skin but also repositions underlying tissues, resulting in a more natural and refreshed appearance. Additionally, reconstructive rhytidectomy can effectively address specific facial concerns, such as jowls and deep nasolabial folds, creating a more balanced and harmonious facial contour. The long-lasting results of this surgery can help maintain a youthful appearance for many years, making it a valuable option for those looking to counteract the signs of aging.

A rhytidectomy can aid in achieving facial symmetry, but it is typically not performed concurrently with dynamic reanimation procedures. Instead, it is more commonly applied to the contralateral healthy side, as the reconstructed side is generally augmented with a muscle flap, resulting in a younger and fuller appearance [28]. According to the American Society for Aesthetic Plastic Surgery, rhytidectomy is one of the most sought-after surgical cosmetic procedures. In 2017, it was the sixth most common procedure among women and the fifth among men, with over 80,000 surgeries conducted. Given its ongoing popularity, it is essential to identify, prevent, and address the well-documented complications associated with facelift surgery [29].

A prospective cohort study involving 126,713 patients conducted between 2008 and 2013 indicates that reconstructive rhytidectomy is a highly safe procedure when performed by board-certified plastic surgeons [30]. There is substantial supportive evidence for the use of preinfiltration in facelift surgery, indicating that fat grafting and laser skin resurfacing can be safe and effective adjuncts for appropriately selected patients. Additionally, while the use of drains is not mandatory, it likely provides some benefits [31]. Rhytidectomy can improve disfigurement caused by facial volume loss by tightening the skin. One case report documented a patient with HIV-associated facial lipoatrophy who underwent a limited facelift, utilizing a pre-auricular incision along with a horizontal incision in the sideburn area. Both the patient and the surgeon expressed satisfaction with the results; however, there is a lack of long-term follow-up data [32].

Generally, facelift procedures can significantly enhance facial appearance by reducing signs of aging, such as sagging skin and deep wrinkles, leading to a more youthful and revitalized look. The procedure not only improves facial contours but also boosts self-confidence and overall satisfaction with one's appearance. Additionally, a facelift can offer long-lasting results, helping individuals maintain a fresher, more invigorated appearance for years to come.

Disadvantages

While reconstructive rhytidectomy offers several advantages, it also presents potential drawbacks that individuals should consider. One major concern is the risk of complications, including infection, scarring, or adverse reactions to anesthesia. The recovery period can be lengthy and uncomfortable, often requiring considerable downtime before resuming normal activities. Additionally, the results may not be permanent, as the natural aging process continues, which could necessitate further procedures in the future. There is also a chance of dissatisfaction with the aesthetic outcome, potentially leading to emotional distress. Lastly, the financial cost of the surgery can be significant, and it is typically not covered by insurance, making it a considerable investment for many individuals. Recent research demonstrates that the most frequent major complications are hematoma and infection. Additionally, male gender, a BMI of 25 kg/m^2^ or higher, and undergoing combined procedures are identified as independent risk factors [33]. As with other cosmetic surgeries, the most frequent negative outcome is dissatisfaction with the aesthetic results, which can stem from various factors such as scarring, asymmetry, contour irregularities, or an over- or under-corrected appearance. Establishing a strong rapport with the patient before the surgery can assist the surgeon in navigating any postoperative challenges that may arise, potentially enhancing patient satisfaction and reducing the risk of legal action in the event of an unsatisfactory outcome [34].

The relative novelty of numerous anatomical descriptions, coupled with inconsistent terminology and the natural variability in tissue morphology, makes facelift procedures a challenging subject for many young plastic surgeons. This complexity is exacerbated by the wide range of surgical techniques available, each often claiming to achieve a "successful" outcome. A thorough understanding of fundamental anatomy and a deep appreciation of the principles of facial aging are essential for grasping the various facelift techniques effectively [35].

A study analyzed the incidence and risk factors for major complications following facelift procedures using a large, prospective, multicenter database. It specifically compared complications occurring in facelifts performed alone versus those combined with other cosmetic surgeries. The research identified a prospective cohort of patients who underwent facelifts between 2008 and 2013, sourced from the CosmetAssure database. Out of 129,007 patients enrolled in this database, 11,300 (8.8%) had facelifts. The findings revealed that facelifts were associated with a complication rate of 1.8%, which is comparable to the 2% complication rate linked to other cosmetic surgeries. The most frequently observed issues were hematomas at 1.1% and infections at 0.3%. Furthermore, procedures that were combined with facelifts had a higher complication rate of up to 3.7%, compared to 1.5% for facelifts performed in isolation [36].

Future Directions

Reconstructive rhytidectomy, also known as facelift surgery, is evolving due to advancements in surgical techniques and technology. Future developments will likely emphasize minimally invasive procedures, employing endoscopic methods to minimize scarring and shorten recovery times. The incorporation of regenerative medicine, such as stem cell therapy and platelet-rich plasma, may further enhance tissue healing and rejuvenation. Personalized strategies, including 3D imaging and virtual simulations, will enable tailored surgical plans that cater to individual patient needs. Additionally, a focus on holistic care will encourage the integration of surgical and noninvasive treatments, promoting comprehensive facial rejuvenation and greater patient satisfaction.

Current trends in facial rejuvenation highlight the rising popularity of noninvasive skin tightening techniques, including radiofrequency (both monopolar and bipolar), microfocused ultrasound, and general laser bulk heating. These modalities work by delivering heat to the deep dermis and fascia, which helps contract existing collagen and stimulate the production of new collagen. While these techniques represent innovative options in the field, their long-term efficacy remains to be fully understood. Nonetheless, they present a noninvasive alternative that typically yields modest results compared to surgical facelifts, whose effects tend to diminish after 1.5-2 years [36]. Interestingly, ancillary procedures used during facelifts include dermabrasion, ablative lasers, chemical peels, and radiofrequency devices. All these techniques are safe and effective, and they should be customized according to the patient's anatomy, expectations, and tolerance for side effects and healing time. Over the past 47 years of utilizing each ancillary modality, the senior author has noted a trend toward a facelift demographic that prefers shorter recovery times and lower risks of complications. Radiofrequency devices have adapted to this demand, offering a high level of safety, reliability, and reproducibility. Treatments such as Morpheus8 and FaceTite/AccuTite have entered common usage, with many patients readily explaining the mild erythema by saying, "I had Morpheus." Currently, Morpheus8 remains the most frequently performed ancillary procedure in the authors' practice, and its role in primary nonsurgical facial rejuvenation continues to expand [37].

The field of facial rejuvenation is dynamic and evolving, with facelift techniques evolving from minimally invasive short flap approaches to more aggressive deep plane techniques, and now back to minimally invasive methods that consider volumization. A significant advancement over the past 40 years is the customization of techniques to suit each individual patient, focusing on the specific signs of aging that need to be addressed. Today, a variety of techniques are available and can be tailored to meet the unique desires and anatomical features of each patient.

Recent research reported that the combination of rhytidectomy (facelift) and fat grafting is a safe and effective approach to simultaneously treat age-related ptosis and volume loss [38]. Similarly, it was indicated recently that effective facelifting relies on understanding and addressing the comprehensive changes that occur with aging. This includes recognizing facial asymmetry and employing suitable techniques to treat each side of the face individually, while also considering the overall aesthetic balance. Although the methods outlined in this article are based on expert opinion and experience, we have observed that facelift outcomes improve when asymmetries are identified and incorporated into the surgical plan. While individual results may vary in duration, incorporating fat grafting typically extends the longevity of facelift results. While achieving perfect facial symmetry is neither possible nor advisable, striving for it can enhance facial harmony, particularly as aging affects each side of the face differently [39].

## Conclusions

Reconstructive rhytidectomy is a highly adaptable surgical procedure that serves a dual purpose: it enhances cosmetic appearance while also facilitating functional reconstruction. This versatility allows it to address a wide range of patient needs, from aesthetic improvements to restoring facial function after trauma or disease. Recent advancements in surgical techniques and technologies have significantly transformed the landscape of reconstructive rhytidectomy. Innovations such as laser surface scanning enable surgeons to create precise, three-dimensional maps of the facial structure, allowing for more tailored and effective interventions. Additionally, the use of tissue sealants has streamlined the surgical process, promoting faster healing and minimizing complications. To achieve optimal results, it is essential to adopt patient-specific approaches that consider individual anatomical variations and aesthetic goals. A comprehensive understanding of facial anatomy is crucial, as it informs the surgical strategy and helps predict outcomes. By integrating these advanced techniques with a personalized approach, surgeons can enhance both the functional and aesthetic aspects of the face, ultimately leading to improved patient satisfaction and quality of life.
